# Supplementary material for: Ultrahigh strength and shear-assisted separation of sliding nanocontacts studied in situ
Source: Nat Commun. 2022 May 10;13:2551. doi: 10.1038/s41467-022-30290-y (PMC9091249; doi:10.1038/s41467-022-30290-y)
Supplement: Supplementary file 1 — Supplementary Information [file 41467_2022_30290_MOESM1_ESM.pdf]

Supplementary Information for
Ultrahigh Strength and Shear-Assisted Separation of Sliding Nanocontacts Studied *in situ*

Supplementary Discussion 1 (Calculation to estimate e-beam damage of the specimen)

The effect of the TEM electron beam was evaluated and shown to be negligibly small.

First we consider the displacement energy. When the energy $\boldsymbol{E_e}$ as described in below equation exceeds a specific threshold energy $\boldsymbol{E_d}$, the electron beam can displace atomic nuclei to interstitial positions [1].

$$E_e=E_0(1.02+E_0/10^6)/(465.7A) \tag{1}$$

where the incident-electron energy E_0 of our TEM was 200 keV, and atomic weight A of Silver was 107.9. The energy E_e from the electron beam is calculated as 4.86 eV. The threshold energy E_d of silver is 25 eV [2,3] and it is 5 times higher than the energy due to the electron beam of TEM. Therefore, it cannot displace atomic nuclei to interstitial positions and thereby the beam does not degrade the crystalline perfection of the silver part. We concluded that the experimental aberrations due to the displacement energy can be considered negligible.

The current flowing on the surface of the specimen j_{sample} was calculated as below. We estimated the current flowing on the surface of the tip when the electron beam illuminates the specimen. The current density j_{all}, when the beam was enlarged up to 11 cm in diameter, was experimentally measured. This density was found to be

$$j_{all}= 0.5\times10^{-11} \quad [\mathrm{A/cm^2}] \tag{2}$$

The current density j', when the beam was narrowed down to 4cm in diameter, was calculated as below

$$j' = j_{all}\,\{\pi\,(11/2)^2\}\;/\;\{\pi\,(4/2)^2\}$$

$$= 4.5\times10^{-11} \qquad [\mathrm{A/cm^2}] \qquad (3)$$

The current density j_{sample}, which was applied to the specimen, was calculated as below.

$$j_{sample} = j'\,(3\mathrm{nm}\times1000{,}000)^2$$

$$= 4.05\times10^{-12}\ [\mathrm{A}]$$

$$= 4.05 \qquad [\mathrm{pA}] \qquad (4)$$

Therefore, we calculated the current flowing on the 3nm square specimen at 1,000,000X magnification as 4.05 pA and found the surface current was negligibly small. Furthermore, since both tips are connected to GND, no charge accumulation occurs within the sample specimen.

The increase in the temperature ΔT due to the e-beam was calculated as below. According to the reference [4],

$$H=Q'J \qquad (Q'=\rho Q) \qquad (5)$$

$$Q=Q_c+Q_r \qquad (6)$$

$$K\nabla^2 T=H \qquad (7)$$

where, H is the energy transfer rate, Q is the average energy loss of an electron per unit length, J is the electron current density which is experimental obtained as 4.5×10^{-11} [A cm^{-2}], Q_c is the Collision stopping power, 1.671 [MeV cm^2 g^{-1}], Q_r is the radiative stopping power, 0.03201 [MeV cm^2 g^{-1}], ρ is the mass density of silver, 10500 [Kg m^{-3}], k is the thermal conductivity of silver 420[W m^{-1} K^{-1}], the values of Q_c, Q_r for Ag were obtained by reference [5]. And then

$$H=QJ$$

$$=(1.671+0.03201)\times 10500\times 4.5\times 10^{-11} \quad [\mathrm{MeV\ cm^{2}\ g^{-1}\ Kg\ m^{-3}\ A^{-1}\ A\ cm^{-2}}]$$

$$=8.037\times 10^{-4} \quad [\mathrm{eV\ cm^{-3}}]$$

$$=1.286\times 10^{-22} \quad [\mathrm{J\ cm^{-3}}] \qquad (8)$$

$$\nabla^{2}T=(1.286\times 10^{-22})/(4.2) \quad [\mathrm{J\ cm^{-3}}]/[\mathrm{J\ s^{-1}\ cm^{-1}\ K^{-1}}]$$

$$=3.062\times 10^{-7} \quad [\mathrm{K\ s\ nm^{-2}}] \qquad (9)$$

The increase in the temperature, ΔT, when we shine the electron beam on the 1nm square specimen for one minute is therefore

$$\Delta T=3.062\times 10^{-7}\times 60$$

$$=18 \quad [\mu\mathrm{K}] \qquad (10)$$

As such the heating rate is approximately one milikelvin per hour of observation, and therefore the increase in temperature due to the electron beam is negligibly small.

Supplementary Discussion 2 (Uncertainty analysis)

The values of the error were calculated as follows. We performed similar experiments for six trials. One of the trials was described in the main manuscript. The other five experiments were described in the Supplementary data. In this section, error value is discussed. The error in sliding distance in the lateral axis Δx depends on the clarity of TEM images because the sliding distance of the tip was measured by marking a characteristic feature on the tip and tracking the feature. Although the TEM resolution is 0.1 nm, the outline of the tip has a slightly larger width, and it is impossible to precisely measure the displacement less than the thickness of the outline. The width of the outline depends on the magnification of TEM images and the values were found to be 0.2 nm.

$$\Delta x = 0.2 \text{ [nm]} \quad (11)$$

The friction force ΔF error was derived by the propagation of the error of the stiffness Δk_x and the error of the sliding distance Δx, because the force was calculated as the product of the rigidity of spring constant k and the displacement of the tip x.

$$\Delta F = \sqrt{(\frac{dF}{dk_x}\Delta k_x)^2 + (\frac{dF}{dx}\Delta x)^2} \quad (12)$$

$$\frac{dF}{dk_x} = x \qquad (\because F = k_x x) \quad (13)$$

k_x was obtained by the measurement of the resonant frequency. It is possible to precisely read the peak of the resonant frequency with 10 Hz (Δf) accuracy. Therefore, the error of the stiffness Δk_x is as below.

$$\Delta k_x = \left(\frac{dk_x}{df_x}\right)\Delta f$$

$$\therefore \Delta k_x = \left(\frac{23}{200}\right)\frac{M f_x \Delta f}{\pi^2} \qquad (\because f_x = 2\pi\sqrt{\frac{k_x}{0.23M}}\ ) \quad (14)$$

(13), (14) give that

$$\frac{dF}{dk_x}\Delta k_x = x\left(\frac{23}{200}\right)\frac{Mf_x\Delta f}{\pi^2} \tag{15}$$

$$\frac{dF}{dx} = k_x \qquad (\because F = k_x x) \tag{16}$$

(16) gives that

$$\frac{dF}{dx}\Delta x = k_x\Delta x \tag{17}$$

Substituting (15) and (17) to (12),

$$\Delta F = \sqrt{\{x(\frac{23}{200})\frac{Mf_x\Delta f}{\pi^2}\}^2 + (k_x\Delta x)^2}$$
$$= 0.6\ (\mathrm{nN}) \tag{18}$$

Therefore the friction force error ΔF was derived as 0.6 nN. The displacement of the beam x is 3.8×10^{-9} m, the mass of the beam M is 8.0×10^{-10} kg, the resonant frequency (experimental data) f_x is 6470 Hz, the error of the measurement Δf is 10 Hz, the stiffness of the beam k_x is 3.1 N/m, the error of the displacement Δx is the results of Eq.11.

The load error ΔL was derived through a similar procedure to that used to measure the error ΔF.

$$\Delta L = \sqrt{(\frac{dL}{dk_y}\Delta k_y)^2 + (\frac{dL}{dy}\Delta y)^2}$$

$$= \sqrt{\{y(\frac{23}{200})\frac{Mf_y\Delta f}{\pi^2}\}^2 + (k_y\Delta y)^2}$$
$$= 6\ (\mathrm{nN}) \tag{19}$$

Therefore the load error ΔL was derived as 6 nN. The displacement of the beam x is 3.8×10^{-9} m,

the mass of the beam M is 25.8×10^{-10} kg, the resonant frequency (experimental data) f_y is 10944 Hz, the error of the measurement Δf is 10 Hz, the stiffness of the beam k_y is 30 N/m, the error of the displacement Δx is the results of Eq.11.

The shear force ΔF_{shear} error was calculated as below. The shear force F_{shear} and the normal force F_{normal} were obtained by Eq.20 where F is friction force, L is load, and θ is the angle between the direction of actuation and the plane of contact.

$$\begin{pmatrix} F_{shear} \\ F_{normal} \end{pmatrix} = \begin{pmatrix} cos\theta & -sin\theta \\ sin\theta & cos\theta \end{pmatrix} \begin{pmatrix} F \\ L \end{pmatrix} \tag{20}$$

Therefore, the shear force error ΔF_{shear} was

$$\Delta F_{shear} = \sqrt{(\frac{dF_{shear}}{dF}\Delta F)^2 + (\frac{dF_{shear}}{dL}\Delta L)^2 + (\frac{dF_{shear}}{d\theta}\Delta\theta)^2}$$

$$= \sqrt{(\Delta F cos\theta)^2 + (\Delta L sin\theta)^2 + (\Delta\theta)^2(F sin\theta + L cos\theta)^2}$$

$$= \sqrt{(0.6 \times cos14.4)^2 + (6.0 \times sin14.4)^2 + (0.052)^2(11.82 \times sin14.4 + 18.19 \times cos14.4)^2}$$

$$= 1.93 \text{ (nN)}$$

Therefore the error of shear force ΔF_{shear} was calculated to be 1.93 nN. The of friction force error ΔF is 0.6 nN, the load error ΔL is 6.0 nN, the value of the contact angle just prior to the separation θ: 14.4°, the value of friction force just prior to the separation F is 11.82 nN, the value of load just prior to the separation L is -18.19 nN, the contact angle error just prior to the separation $\Delta\theta$ is 4.0°.

The normal force error ΔF_{normal} was derived through a similar procedure to that used to measure the error ΔF_{shear}.

$$\Delta F_{normal} = \sqrt{(\frac{dF_{normal}}{dF}\Delta F)^2 + (\frac{dF_{normal}}{dN}\Delta N)^2 + (\frac{dF_{normal}}{d\theta}\Delta\theta)^2}$$

$$= \sqrt{(\Delta F sin\theta)^2 + (\Delta L cos\theta)^2 + (\Delta\theta)^2(F cos\theta - L sin\theta)^2}$$

= 5.83 (nN)

Therefore, the normal force error ΔF_{normal} was derived as 5.83 nN. Where the friction force error ΔF is 0.6 nN, the load error ΔL is 6.0 nN, the value of the contact angle just prior to the separation θ is 14.4°, the value of friction force just prior to the separation F is 11.82 nN, the value of load just prior to the separation L is 18.19 nN, the contact angle error just prior to the separation $\Delta\theta$ is 4.0°.

The value of the contact width w was measured by detecting both side of the junction. Therefore, the resolution Δx ($=\Delta y$) affects the contact width of the junction error Δw.

$$\Delta w = \sqrt{(\Delta x)^2 + (\Delta x)^2}$$

$$= \sqrt{(0.3)^2 + (0.3)^2}$$

= 0.29 [nm]

The value of the von Mises stress S_{vM} was calculate by Eq.21. F_{shear} was shear force calculate by Eq.22. F_{normal} was the normal force calculate by Eq.23. and A was the actual contact area calculated by Eq.24 where w was the value of contact width.

$$S_{vM} = \sqrt{\sigma^2 + 3\tau^2} \quad (21)$$

$$\tau = \frac{F_{shear}}{A} \quad (22)$$

$$\sigma = \frac{F_{normal}}{A} \quad (23)$$

$$A = \pi(\frac{w}{2})^2 \quad (24)$$

Therefore the von Mises stress error ΔS_{vM} was calculate by Eq.25.

$$\Delta S_{vM} = \sqrt{(\frac{dS_{vM}}{d\sigma}\Delta\sigma)^2 + (\frac{dS_{vM}}{d\tau}\Delta\tau)^2}$$

$$= \sqrt{\left(\frac{\sigma^2}{\sigma^2+3\tau^2}\Delta\sigma^2\right) + \left(\frac{9\tau^2}{\sigma^2+3\tau^2}\Delta\tau^2\right)}$$

$$(\because \frac{dS_{vM}}{d\sigma} = \frac{\sigma}{\sqrt{\sigma^2+3\tau^2}}, \frac{dS_{vM}}{d\tau} = \frac{3\tau}{\sqrt{\sigma^2+3\tau^2}}) \tag{25}$$

According to Eq.23,

$$\Delta\sigma = \sqrt{(\frac{d\sigma}{dF_{normal}}\Delta F_{normal})^2 + (\frac{d\sigma}{dA}\Delta A)^2}$$

$$= \sqrt{(\frac{\Delta F_{normal}}{A})^2 + (\frac{F_{normal}}{A^2}\Delta A)^2} \quad (\because \frac{d\sigma}{dF_{normal}} = \frac{1}{A}, \frac{d\sigma}{dA} = \frac{-F_{normal}}{A^2}) \tag{26}$$

According to Eq.22,

$$\Delta\tau = \sqrt{(\frac{d\tau}{dF_{shear}}\Delta F_{shear})^2 + (\frac{d\tau}{dA}\Delta A)^2}$$

$$= \sqrt{(\frac{\Delta F_{shear}}{A})^2 + (\frac{F_{shear}}{A^2}\Delta A)^2} \quad (\because \frac{d\tau}{dF_{shear}} = \frac{1}{A}, \frac{d\tau}{dA} = \frac{-F_{shear}}{A^2}) \tag{27}$$

According to (14), the actual contact area error is

$$\Delta A = \frac{dA}{dw}\Delta w$$

$$= \frac{\pi}{2} w\Delta w$$

$$= 2.15 \text{ [nm]} \tag{28}$$

Substituting (26), (27), (28) to (25),

$$\Delta S_{vM} = 0.29 \text{ [GPa]}$$

where the shear force error ΔF_{shear} is 1.93 nN, the value of shear force F_{shear} is 6.94 nN, the normal force error ΔF_{normal} is 5.83 nN, the value of normal force F_{normal} is -20.6 nN, the value of actual contact area just

prior to the separation A is 11.8 nm^2, the value of shear stress just prior to the separation: 0.38 GPa, the value of normal stress just prior to the separation is -1.11 GPa, the contact width just prior to the separation w is 0.29 nm. The uncertainty values of all experiments were derived from similar procedures above.

Supplementary Discussion 3 (Calculation of the critical contact width)

Aghababaei *et al.* [6] calculated a critical contact width for this process to occur in shearing asperities based on the increase in the surface energy produced by fracture and the work done by external forces due to shear stress. If the contact width is smaller than the critical contact width, asperity fracture and wear debris occurs. The critical contact width $d*$ is derived from Eq.(29).

$$d^* = \frac{G\Delta w}{\sigma^2} \tag{29}$$

where Δw is the work adhesion of silver as 2.6 (J/m^2) (see discussion below), G is the shear modulus as 27.8 (GPa), σ_j is the shear stress at separation. The experimental data of shear stress at separation are 0.38, 0.36, 0.41, 0.37, 0.10, and 0.14 (GPa). Thus, each value of the critical contact width is 1528, 1686, 1286, 1604, 20891, and 11089 (nm). And the contact width obtained from TEM images are 5.46, 4.56, 7.21, 7.12, 10.69, and 8.96 respectively. It was found that the critical contact widths were 170-1900 times larger than the values of the contact width obtained from the experiments. Thus, the model of Aghababaei *et al.* predicts no asperity fracture, consistent with our results.

Supplementary Discussion 4 (Work of adhesion of the asperities)

Analysis of our experiments using contact mechanics modeling requires an estimate of the work of adhesion, which in turn depends on the crystal orientation of the contacting surfaces. Unfortunately, we are not able to determine the crystal orientation of the asperities. The TEM images show mostly striped patterns; we occasionally see regions of limited size with individual atomic columns resolved (which would appear as isolated dots in the TEM image). Regardless, without optimizing via double tilting of the TEM specimen and/or the use of diffraction (neither of which were available for this experiment), it is not possible to unambiguously determine the crystal orientation of the surfaces of the two asperities.

Furthermore, our experiments are distinct from sliding at a single crystal interface, or the fracture of a nanowire, because the rounded asperity's motion involves tracing over the other rounded asperity. Therefore, the two crystal orientations at the interface change as sliding occurs. It is thus not appropriate to specify a specific description of the interfacial structure. Rather, we consider contact to as a bicrystal interface with proximal free surfaces, where we use the orientation-averaged surface and grain boundary energies of Ag as a guide.

When two different crystal surfaces 1 and 2 of the same material form an interface, the work required to separate them is expressed as $W = \gamma_1 + \gamma_2 - \gamma_{GB}$, where γ_{GB} is the grain boundary energy. The surface energy of Ag has been reported to have moderate anisotropy; an orientation-averaged value in experimental studies of approximately 1.3 J/m^2 has been consistently reported and matches with the values obtained for various crystal plans determined by first principles calculations [7-11]. Accordingly, we assume that $\gamma_1 = \gamma_2 = 1.3$ mJ/m^2. An orientation-averaged value for the grain boundary energy of Ag was reported in experiments to be 0.480±0.109 J/m^2 [12]. Udler and Seidman used embedded atom potentials to calculate the energies for five different low and high angle grain boundaries in Ag, obtaining values ranging from 0.55-0.75 J/m^2 [13]. Recent density functional theory calculations of the grain boundary energy for 10 different grain boundaries in Ag had an average value of 0.5 J/m^2, while a recent calculation of

a comprehensive set 200 grain boundaries in Ag using the embedded atom model gave values ranging from 0.5-1.0 J/m^2. Based on these results, we choose $\gamma_{GB} = 0.6$ J/m^2 as a reasonable estimate. These assumed values lead to W=2.0 J/m^2. We caution that, due to the uncertainty in orientation of our asperity surfaces and the range of values in the literature, this is only an approximate value. However, we have found that varying W by ±20% still requires us to invoke a significant influence of the shear force to explain the observed the pull-off forces.

Similar experiments were performed for six trials in total. Each experiment led to a different contact width.

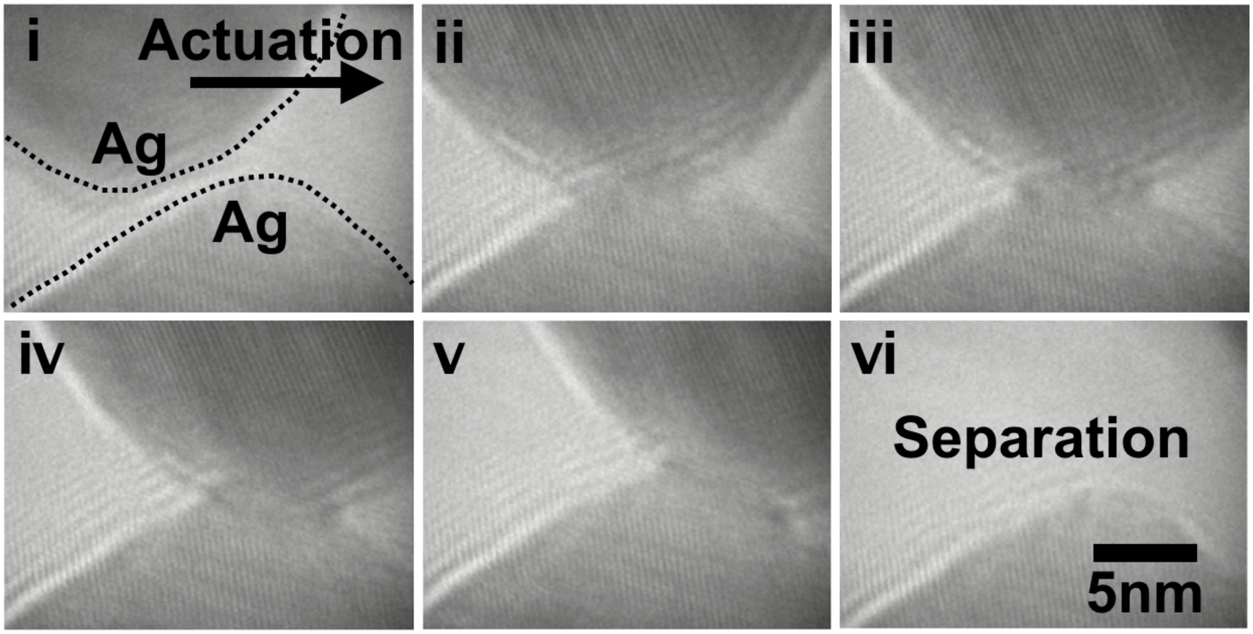

Supplementary Fig. 1 | TEM images of single nano-asperity contacts that was different contact width. **i,** The upper tips was actuated in the lateral direction. **ii,** Two opposing tips were brought into contact. **iii, iv, v,** The upper tip was drugged across the other tips. **vi,** the junction was separated.

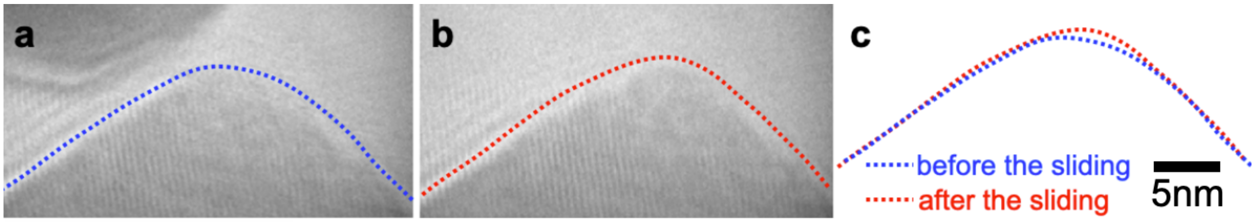

Supplementary Fig. 2 | TEM images demonstrate that nanoscale plastic deformation occurred due to contact and separation. The shape before the contact as shown in **a** was compared with the shape after the separation as shown in **b**. **c** depicted the difference. TEM experiment demonstrated that the angular asperity rounded after the contact separation.

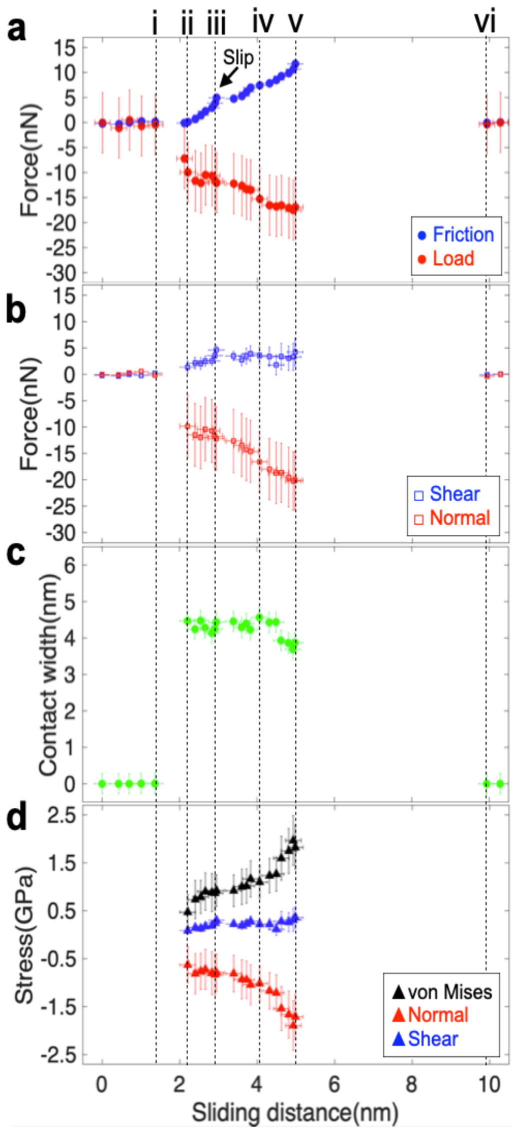

Supplementary Fig. 3 | Forces (from the NEMS device), contact width (from TEM images), and resulting calculated stresses as a function of sliding distance. a, Friction force and load are plotted. **b,** The shear forces and normal forces are plotted. **c,** the contact width was measured as the shortest length of the junction. **d,** The von Mises stress and the normal stress were plotted. **i-vi** in the graph of **a-d** corresponds to **i-vi** in fig.S.1. The error bars representing the uncertainty of each experimental value arose from the resolution of the TEM and NEMS actuator, and the calculations are performed as described in Supplementary Discussion 2.

Another experiment was performed with different actual contact area.

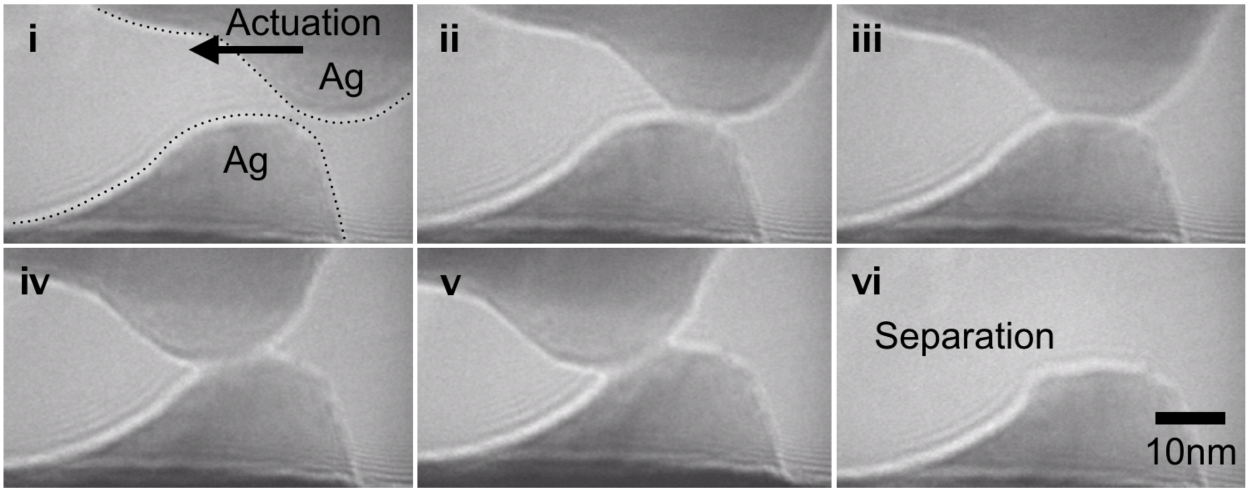

Supplementary Fig. 4 | TEM images of single nano-asperity contacts that was different contact width. i, The upper tips was actuated in the lateral direction. **ii,** Two opposing tips were brought into contact. **iii, iv, v,** The upper tip was dragged across the other tips. **vi,** the junction was separated.

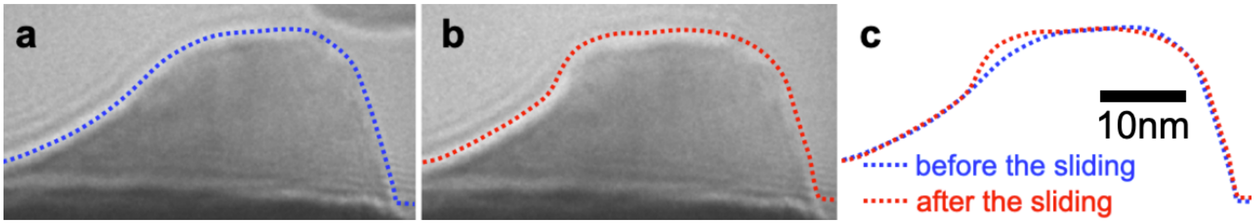

Supplementary Fig. 5 | TEM images demonstrate that nanoscale plastic deformation occurred due to contact and separation. The shape before the contact as shown in **a** was compared with the shape after the separation as shown in **b**. **c** depicted the difference. TEM experiment demonstrated that the angular asperity rounded after the contact separation.

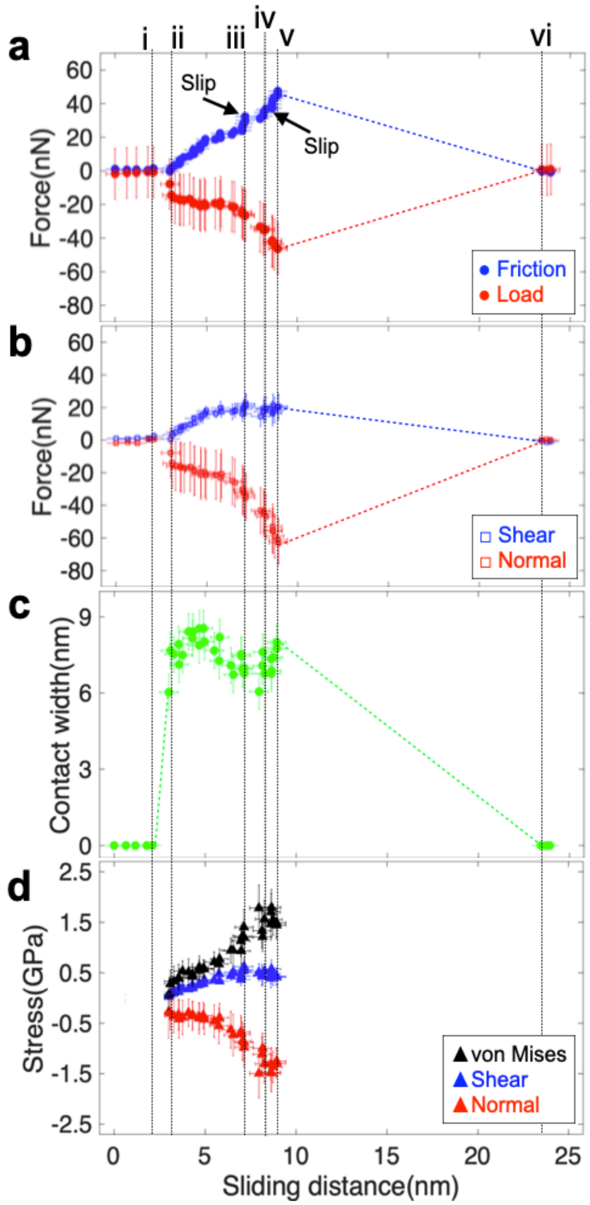

Supplementary Fig. 6 | Forces (from the NEMS device), contact width (from TEM images), and resulting calculated stresses as a function of sliding distance. a, Friction force and load are plotted. **b,** The shear forces and normal forces are plotted. **c,** the contact width was measured as the shortest length of the junction. **d,** The von Mises stress and the normal stress were plotted. **i-vi** in the graph of **a-d** corresponds to **i-vi** in fig.S.4. The error bars representing the uncertainty of each experimental value arose from the resolution of the TEM and NEMS actuator, and the calculations are performed as described in Supplementary Discussion 2.

Another experiment was performed with different actual contact area.

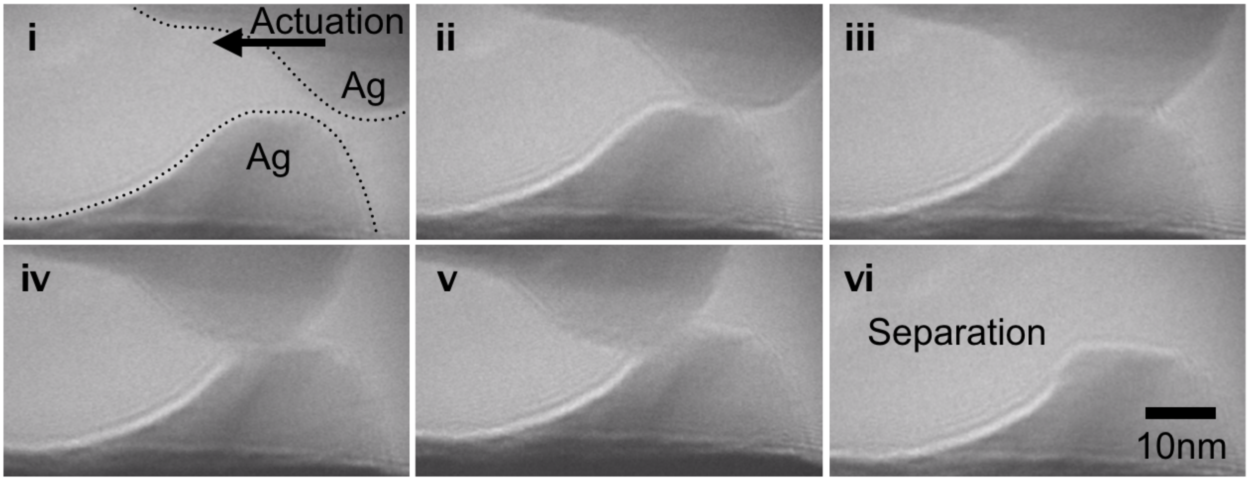

Supplementary Fig. 7 | TEM images of single nano-asperity contacts that was different contact width. **i,** The upper tips was actuated in the lateral direction. **ii,** Two opposing tips were brought into contact. **iii, iv, v,** The upper tip was drugged across the other tips. **vi,** the junction was separated.

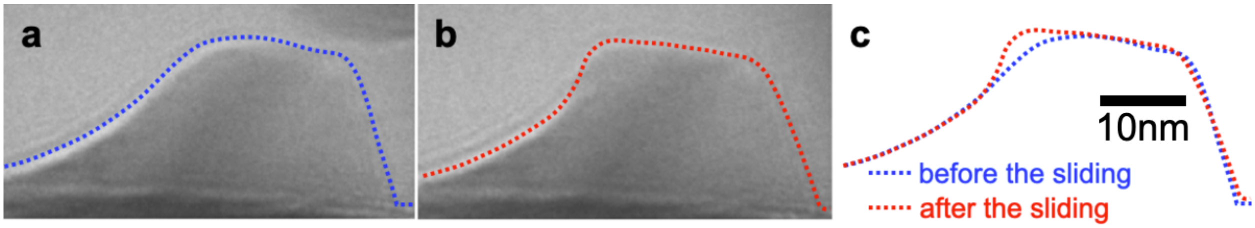

Supplementary Fig. 8 | TEM images demonstrate that nanoscale plastic deformation occurred due to contact and separation. The shape before the contact as shown in **a** was compared with the shape after the separation as shown in **b**. **c** depicted the difference. TEM experiment demonstrated that the angular asperity rounded after the contact separation.

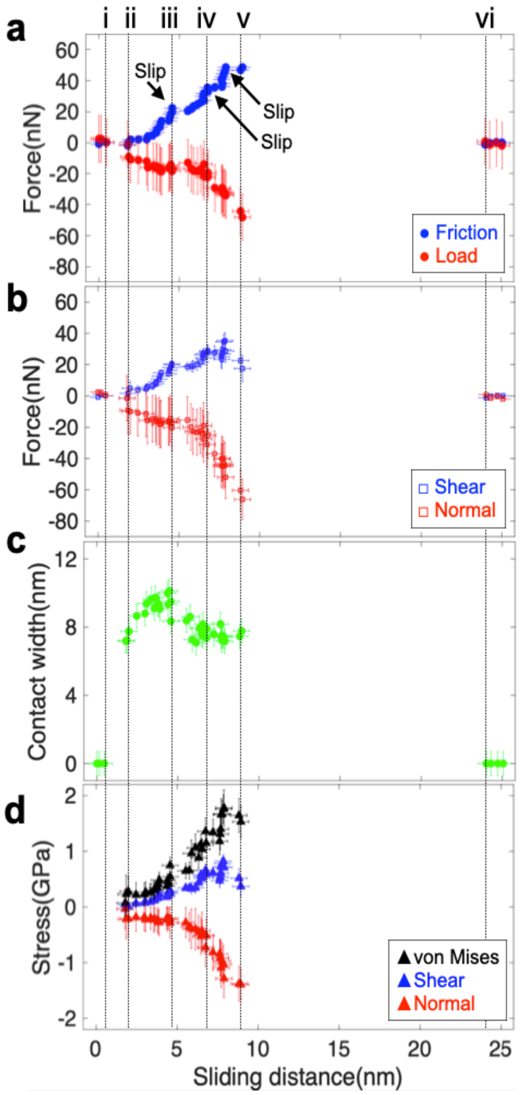

Supplementary Fig. 9 | Forces (from the NEMS device), contact width (from TEM images), and resulting calculated stresses as a function of sliding distance. a, Friction force and load are plotted. **b,** The shear forces and normal forces are plotted. **c,** the contact width was measured as the shortest length of the junction. **d,** The von Mises stress and the normal stress were plotted. **i-vi** in the graph of **a-d** corresponds to **i-vi** in fig. S.7. The error bars representing the uncertainty of each experimental value arose from the resolution of the TEM and NEMS actuator, and the calculations are performed as described in Supplementary Discussion 2.

Another experiment was performed with different actual contact area.

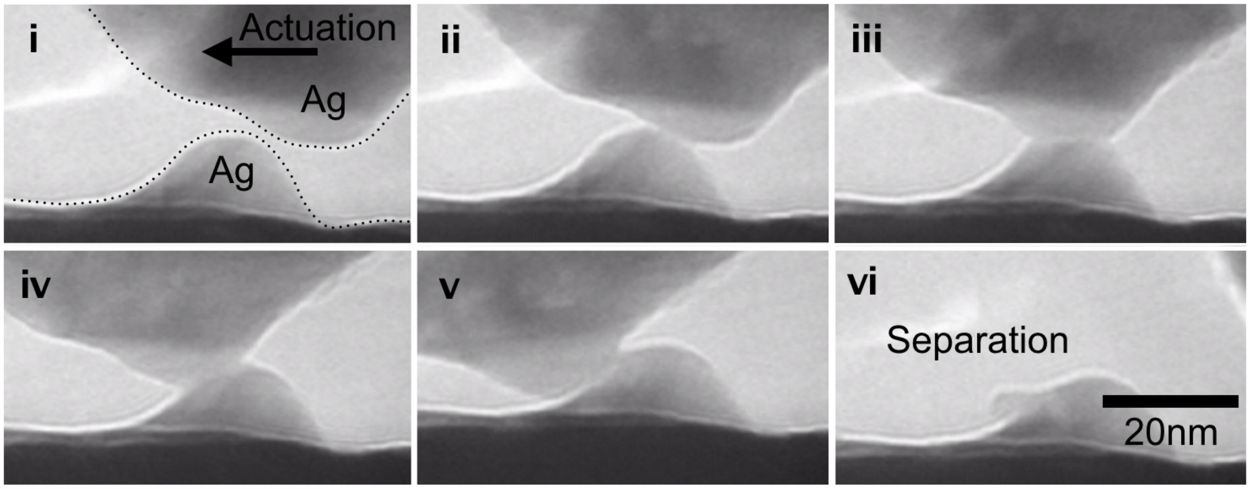

Supplementary Fig. 10 | TEM images of single nano-asperity contacts that was different contact width. i, The upper tips was actuated in the lateral direction. **ii,** Two opposing tips were brought into contact. **iii, iv, v,** The upper tip was drugged across the other tips. **vi,** the junction was separated.

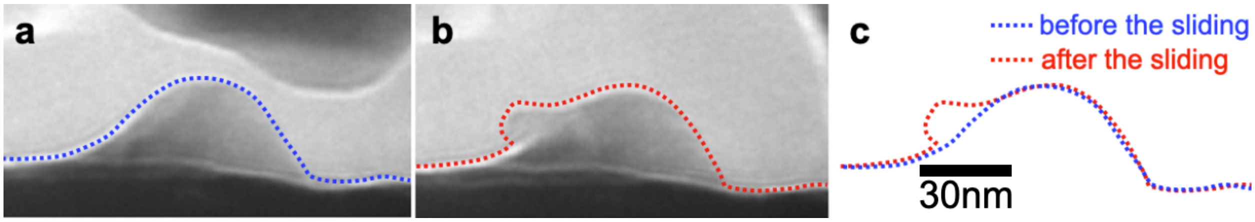

Supplementary Fig. 11 | TEM images demonstrate that nanoscale plastic deformation occurred due to contact and separation. The shape before the contact as shown in **a** was compared with the shape after the separation as shown in **b**. **c** depicted the difference. TEM experiment demonstrated that the angular asperity rounded after the contact separation.

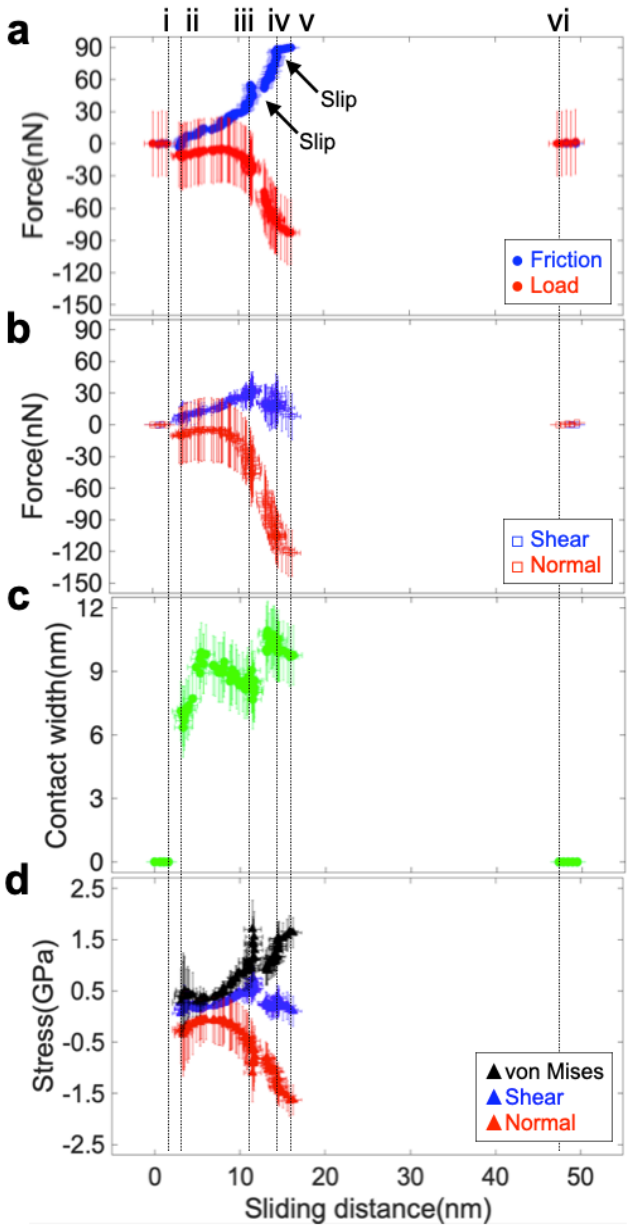

Supplementary Fig. 12 | Forces (from the NEMS device), contact width (from TEM images), and resulting calculated stresses as a function of sliding distance. a, Friction force and load are plotted. **b,** The shear forces and normal forces are plotted. **c,** the contact width was measured as the shortest length of the junction. **d,** The von Mises stress and the normal stress were plotted. **i-vi** in the graph of **a-d** corresponds to **i-vi** in fig. S.10. The error bars representing the uncertainty of each experimental value arose from the resolution of the TEM and NEMS actuator, and the calculations are performed as described in Supplementary Discussion 2.

Another experiment was performed that was different actual contact area.

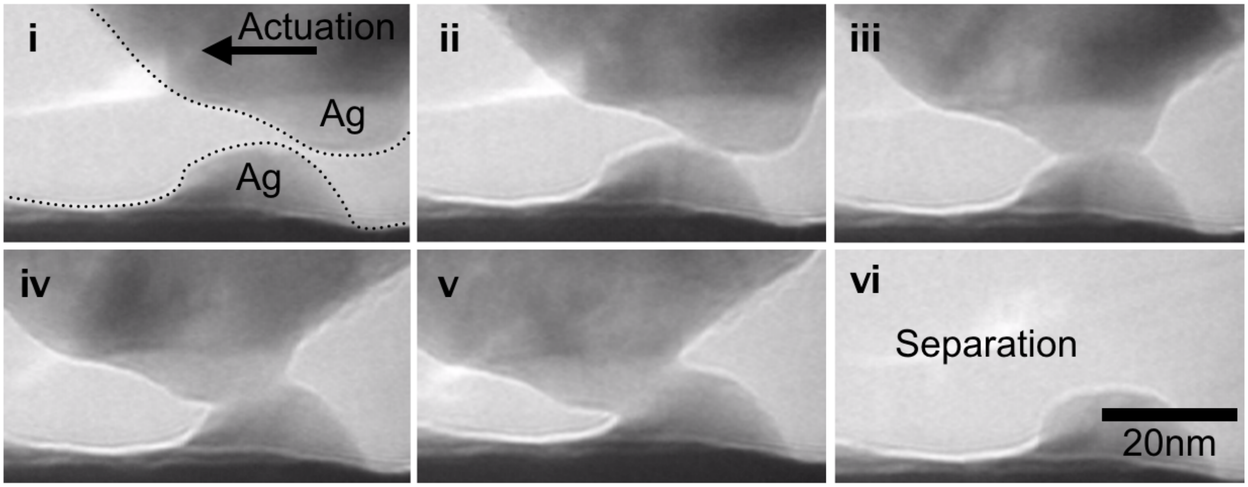

Supplementary Fig. 13 | TEM images of single nano-asperity contacts that was different contact width. i, The upper tips was actuated in the lateral direction. **ii**, Two opposing tips were brought into contact. **iii**, **iv**, **v**, The upper tip was drugged across the other tips. **vi**, the junction was separated.

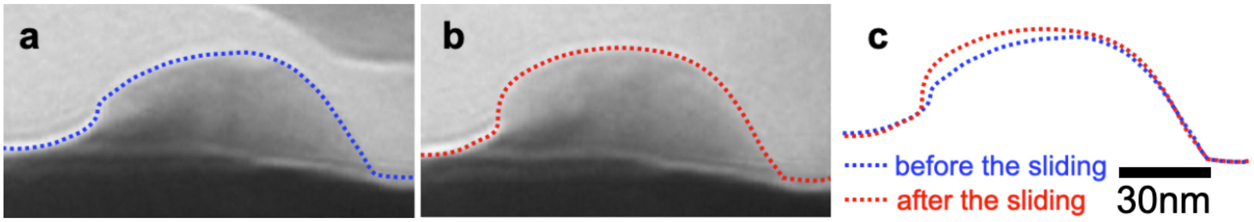

Supplementary Fig. 14 | TEM images demonstrate that nanoscale plastic deformation occurred due to contact and separation. The shape before the contact as shown in **a** was compared with the shape after the separation as shown in **b**. **c** depicted the difference. TEM experiment demonstrated that the angular asperity rounded after the contact separation.

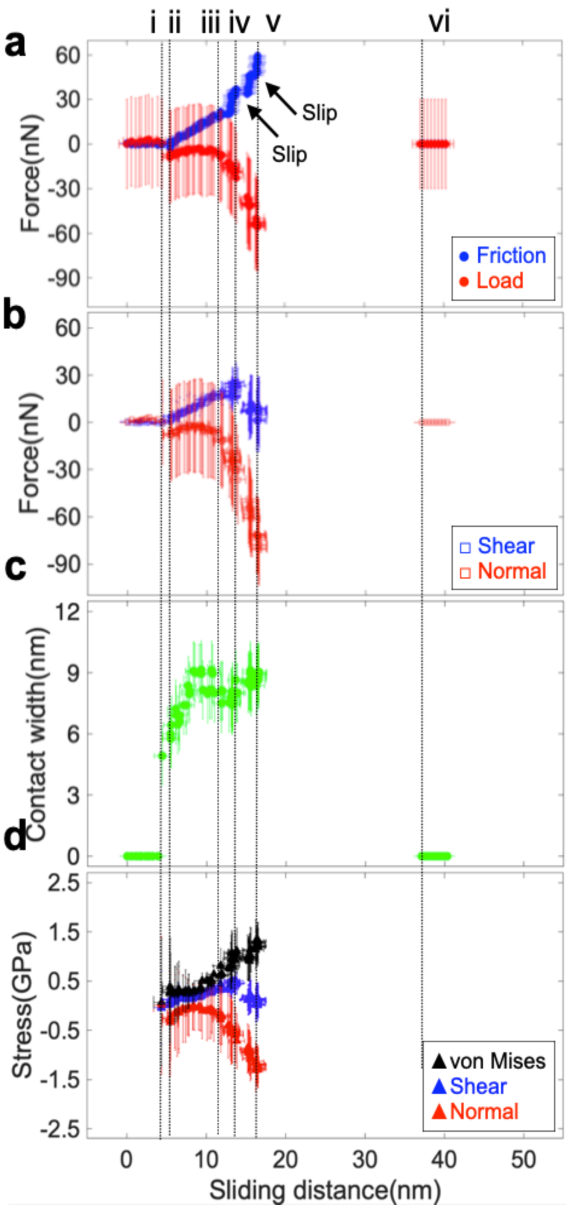

Supplementary Fig. 15 | Forces (from the NEMS device), contact width (from TEM images), and resulting calculated stresses as a function of sliding distance. a, Friction force and load are plotted. **b,** The shear forces and normal forces are plotted. **c,** the contact width was measured as the shortest length of the junction. **d,** The von Mises stress and the normal stress were plotted. **i-vi** in the graph of **a-d** corresponds to **i-vi** in fig. S.13. The error bars representing the uncertainty of each experimental value arose from the resolution of the TEM and NEMS actuator, and the calculations are performed as described in Supplementary Discussion 2.

Supplementary Table. 1 | Unstable sliding property during the lateral actuation. Shear, normal, von Mises, and effective shear stresses at points for each sliding pass in where unstable slip occurred in the six experiments. The sliding distance at which the slip instability occurred, and the measured slip distance along the lateral direction *X* and the overall slip distance *R* (*i.e.*, accounting for lateral and vertical displacement) are also given.

	Sliding pass	Sliding distance (nm)	Slip distance in X (nm)	Slip distance in R (nm)	Shear stress (GPa)	Normal stress (GPa)	von Mises stress (GPa)	Effective shear stress (GPa)
Fig. 3	1st	2.95±0.2	0.52±0.2	0.44±0.2	0.3±0.04	-0.47±0.32	0.70±0.22	0.40±0.13
Fig. S.3	1st	2.94±0.2	0.44±0.2	0.44±0.2	0.3±0.06	-0.76±0.39	0.92±0.33	0.53±0.19
Fig. S.6	1st	6.74±0.4	0.77±0.4	0.77±0.4	0.43±0.17	-1.01±0.39	1.40±0.35	0.81±0.20
	2nd	7.80±0.4	0.34±0.4	0.4±0.4	0.62±0.18	-1.49±0.37	1.78±0.36	1.03±0.21
Fig. S.9	1st	4.56±0.4	0.97±0.4	0.97±0.4	0.37±0.04	-0.37±0.27	0.75±0.15	0.43±0.09
	2nd	6.75±0.4	0.46±0.4	0.51±0.4	0.66±0.1	-0.72±0.34	1.35±0.24	0.78±0.14
	3rd	7.89±0.4	0.92±0.4	0.98±0.4	0.71±0.18	-1.29±0.34	1.78±0.32	1.03±0.18
Fig. S.12	1st	11.5±1.0	1.58±1.0	1.58±1.0	0.75±0.32	-1.09±0.56	1.70±0.56	0.98±0.32
	2nd	14.4±1.0	0.5±1.0	0.52±1.0	0.38±0.23	-1.36±0.31	1.51±0.33	0.87±0.19
Fig. S.15	1st	13.9±1.0	1.3±1.0	1.3±1.0	0.51±0.23	-0.68±0.55	1.12±0.46	0.65±0.27
	2nd	15.7±1.0	0.59±1.0	0.72±1.0	0.2±0.32	-1.06±0.42	1.13±0.44	0.65±0.25

Supplementary Table. 2 | Contact properties at separation. Contact width at separation, asperity radii, Tabor's parameter, experimental pull-off force (force at separation normal to the contact interface), and predicted pull-off forces from the JKR model and the shear index α .

Contact width (nm)	Upper radii (nm)	Lower radii (nm)	Effective probe radii (nm)	Tabor's parameter	Pull-off force (nN) Experiment	Shear force at pull-off (nN) Experiment	von Mises stress at pull-off (GPa) Experiment	Pull-off force (nN) Theory (MD)	MD model using calculated μ_T (nN)	α
4.84±0.28	12.9	6.6	4.37	0.83	20.6±5.8	6.94±1.93	1.29±0.31	57.2	54.9	1.9
3.87±0.28	12	9	5.14	0.88	20.1±5.5	4.23±2.61	1.82±0.46	67.0	64.4	1.9
7.99±0.71	19	18.0	9.24	1.07	62.9±13.4	20.6±7.76	1.44±0.27	118.4	114.4	0.55
7.77±0.71	16.7	16.7	8.35	1.03	66.1±13.0	17.5±8.46	1.53±0.28	107	103.6	0.47
9.77±1.41	26.7	22.9	12.3	1.17	122±21.8	7.7±21.8	1.63±0.29	156	151.6	0
8.91±1.41	20.2	20	10.0	1.10	80.8±22.8	8.73±20.2	1.32±0.37	127	123.7	1.9

Supplementary References

[1] R. F. Egerton, P. Li and M. Malac “Radiation damage in the TEM and SEM” micron, 35, 399-409, 2004

[2] Hobbs, L. W., 1987. Radiation effects in analysis by TEM. In: Hren, J. J., Goldstein, J. I., Joy, D. C. (Eds.), Introduction to Analytical Electron Microscopy, Plenum Press, New York, pp. 399–445.

[3] A. Yu. Konobeyev, U. Fischer, Yu. A. Korovin, S. P. Simakov “Evaluation of effective threshold displacement energies and other data required for the calculation of advanced atomic displacement cross-sections” Nuclear Energy and Technology, 3, 169-175, 2017

[4] Kun Zheng, Chengcai Wang, Yong - Qiang Cheng, Yonghai Yue, Xiaodong Han, Ze Zhang, Zhiwei Shan, Scott X Mao, Miaomiao Ye, Yadong Yin and Evan Ma “Electron-beam-assisted superplastic shaping of nanoscale amorphous silica” Nature Communications, 2010, vol.1, p.24

[5] Berger, M. J., J. H. Hubbell, S. M. Seltzer, J. Chang, J. S. Coursey, R. Sukumar, and D. S. Zucker. "NIST standard reference database." (2010)

[6] Aghababaei R., Warner, D. H. & Molinari, J-F. Critical length scale controls adhesive wear mechanisms, Nature Comm, 7, 11816, (2016).

[7] Skriver. H. K., & Rosengaard, N. M., Surface energy and work function of elemental metals. Phys. Rev. B, 46, 11, (1992)

[8] Vitos, L., Ruban A. V., Skriver, H. L. & Kollar, J. The surface energy of metals. Surface Science, 411, 186-202 (1998)

[9] F. R. de Boer, R. Boom, W. C. M. Mattens, A. R. Miedema, and A. K. Niessen, Cohesion in Metals (North-Holland, Amsterdam, 1988)

[10] Aqra, F. and Ayyad, A. Surface Energies of Metals in Both Liquid and Solid States. Appl. Surf. Sci. 257, 6372-6379 (2011)

[11] Tyson, W.R. and Miller, W.A. Surface Free Energies of Solid Metals: Estimation from Liquid Surface Tension Measurements. Surf. Sci. 62, 267-276 (1977)

[12] Fiala, J., & Ćdek, J. Surface and grain boundary energies of silver at oxygen pressures lower than 10-15 Pa. Philosophical Magazine, 32(1), 251-255. (1975)

[13] Udler, D., & Seidman, D. N. Grain boundary and surface energies of fcc metals. Physical Review B, 54(16), R11133. (1996)
