# Supplementary material for: Ultrahigh strength and shear-assisted separation of sliding nanocontacts studied in situ
Source: Nat Commun. 2022 May 10;13:2551. doi: 10.1038/s41467-022-30290-y (PMC9091249; doi:10.1038/s41467-022-30290-y)
Supplement: Supplementary file 3 — Description of Additional Supplementary Files [file 41467_2022_30290_MOESM3_ESM.pdf]

Description of Additional Supplementary Files

File Name: Supplementary Movie 1
Description: Real-time observation of Ag nano-asperity friction
